# Active sero-survey for European bat lyssavirus type-1 circulation in North African insectivorous bats

**DOI:** 10.1038/s41426-018-0214-y

**Published:** 2018-12-13

**Authors:** Jordi Serra-Cobo, Marc López-Roig, Rachel Lavenir, Elbia Abdelatif, Wahida Boucekkine, Mehdi Elharrak, Bachir Harif, Sehhar El Ayachi, Akram Ahmed Salama, Mohamed A. Nayel, Ahmed Elsify, Sameh G. El Rashedy, Paola De Benedictis, Franco Mutinelli, Barbara Zecchin, Dino Scaravelli, Chokri Balhoul, Ahmed Zaghawa, Hany Youssef Hassan, Ahmed Hamed Zaghloul, Hervé Bourhy

**Affiliations:** 1 IRBIO and Departement de Biologia Evolutiva, Ecologia i Ciències Ambientals, Facultat de Biologia, Universitat de Barcelona, 08028 Barcelona, Spain; 2 Centre de Recerca en Infeccions Víriques, Illes Balears (CRIVIB), Fundació d’Investigació Sanitària de les Illes Balears, Conselleria de Salut i Consum, Govern de les Illes Balears, Hospital General de Palma, 07012 Palma de Mallorca, Illes Balears Spain; 3 Institut Pasteur, Unité Dynamique des Lyssavirus et Adaptation à l’Hôte, WHO Collaborating Centre for Reference and Research on Rabies, Paris Cedex 15, 75724 Paris, France; 4 Institut Pasteur d’Alger, 16047 Dély Ibrahim, Alger Algeria; 5 Direction Générale des Forêts, Chemin Doudou Mokhtar Ben-Aknoun, B.P.232, 16306 Alger, Algeria; 6 Société de Produits biologiques et pharmaceutiques et vétérinaires (Biopharma), Km 2, Road of Casablanca, B.P, 4569 Rabat-Akkari, Morocco; 7 Département Ressources Naturelles et Environnement Institut Agronomique et Vétérinaire Hassan II, Madinat Al Irfane, B.P, 6202 Rabat, Morocco; 8 Department of Animal Medicine and Infectious Diseases, Faculty of Veterinary Medicine, University of Sadat City, 32897 Sadat City, Minoufiya Egypt; 9 Department of Aquatic Animal Medicine, Faculty of Veterinary Medicine, University of Sadat City, 32897 Sadat City, Minoufiya Egypt; 10 FAO and National Reference Centre for rabies & OIE Collaborating Centre for diseases at the animal-human interface, Division of Biomedical Science, Istituto Zooprofilattico Sperimentale delle Venezie, 35020 Legnaro, Italy; 11 S.T.E.R.N.A. & Museo Ornitologico “F. Foschi”, 47121 Forlì, Italy; 12 Laboratory of Pathogens’ Ecology, Department of Veterinary Medical Sciences, University of Bologna, 40064 Ozzano Emilia (Bo), Italy; 13 Institut Pasteur Tunis, Place Pasteur B.P. 74, 1002 Tunis, Belvédère Tunisia; 14 Department of Theriogenology and Artificial Insemination, Faculty of Veterinary Medicine, University of Sadat City, 32897 Sadat City, Minoufiya Egypt

Bats have specific biological and ecological characteristics that make them suitable hosts for viruses; some of these viruses have the ability to infect humans and other domestic and wild mammals^1^. Among these zoonotic viruses, lyssaviruses are known to circulate among the European insectivorous bats^2^. To date, 5 lyssaviruses species and one recently discovered species circulating in Europe have been recognized: *European bat 1 lyssavirus* (EBLV-1), *European bat 2 lyssavirus* (EBLV-2), *Bokeloh Bat Lyssavirus* (BBLV), *Lleida bat lyssavirus* (LLBV), *West Caucasian Bat Lyssavirus* (WVBV) and the tentative novel member of the genus *Lyssavirus Kotalahti* bat lyssavirus^3,4^.

In Africa, to date, three lyssaviruses have been identified in bats: *Lagos bat virus* (LBV), reported for the first time in Nigeria (1956) from an *Eidolon helvum*; *Duvenhage virus* (DUVV), detected in South Africa (1970) from the *Miniopterus* genus; and the *Shimoni bat virus* (SHIBV), isolated from *Hipposideros vittatus* in Kenya (2009)^5^. However, serological evidence of *West Caucasian bat virus* (WCBV) and EBLV-1 infection has been reported in bats from Kenya^6^ and southwestern Indian Ocean islands^7^, respectively. Little is known about the circulation and distribution of insectivorous bat lyssaviruses in North Africa, as well as the impact such viruses may have on public health. Although there is no evidence that bats migrate between the two continents across the Mediterranean Sea^8^, migratory species such as *Miniopterus schreibersii* can fly over the sea for long distances^9^. The aim of this study was to assess the potential circulation of European bat lyssaviruses in Northern Africa from 2007 to 2012.

All bats were handled in strict accordance with good animal practices, as defined by the current European legislation. The capture of bats and blood-sampling were authorized with specific permits issued by the Forest Ministry from Morocco and Algeria.

Sampling sites were selected according to bat behavior and species, which were classified as synanthropic (urban areas), migratory and gregarious. Bat colonies were sampled throughout the year, although hibernation and birthing periods were deliberately avoided. Bats were randomly captured inside the roosts with long-handled butterfly nets in the daytime and with mist nets at sunset, when they emerged to forage, and only in cases where access to the roost interior was not possible. The individuals identified as juveniles were released without further sampling. Morphological identification of bats was performed in compliance with the identification keys for the bats of Europe, and the bats were sexed. Some *Miniopterus* were sampled in a specific location of the Great Atlas, where *Miniopterus maghrebensis* lives. This bat species was described only after our fieldwork had been completed^10^, which means that we had no way to retrospectively determine which individuals belonged to the species *M. maghrebensis*. For this reason, the *Miniopterus* bats sampled in the Atlas region were included in the *Miniopterus* complex reported in Table 1 (M. *maghrebensis*/*M. schreibersii*).

**Table 1:** Serological results of *EBLV-1* analyses from North Africa (2007 to 2012) and comparison with serological results obtained from samples collected in South-West Europe (Spain and Italy) from 2007 to 2015

	North Africa			South West Europe			
Species	Algeria^a^	Morocco^a^	SIR^b^	Spain^a^	Italy^a^	SIR^b^	Total
*F. Rhinolophidae*							
*Rhinolophus ferrumequinum*	1/47	8/67	7.90	38/165	0/15	20.54	15.71
*Rhinolophus euryale*	3/31	0/12	6.98	1/2	nd	50.00	8,89
*Rhinolophus mehelyi*	nd	0/7	0.00	nd	nd	nd	0.00
*Rhinolophus blasii*	0/1	nd	0.00	nd	nd	nd	0.00
*Rhinolophus hipposideros*	nd	nd	nd	nd	0/5	0.00	0.00
*F. Vespertilionidae*							
*Myotis myotis*	nd	nd	nd	127/390	16/205	24.03	24.03
*Myotis blythii*	nd	nd	nd	18/105	11/52	18.47	18.47
*Myotis punicus*	1/63	15/262	4.92	nd	nd	nd	4.92
*Myotis capaccinii*	0/6	nd	0.00	17/99	0/9	15.74	14.91
*Myotis escalerai*	nd	0/1	0.00	6/18	nd	33.33	31.58
*Myotis emarginatus*	0/27	nd	0.00	0/2	nd	0.00	0.00
*Myotis daubentonii*	nd	nd	nd	7/54	nd	12.96	12.96
*Myotis mystacinus*	nd	nd	nd	0/1	nd	0.00	0.00
*Pipistrellus pipistrellus*	nd	nd	nd	7/39	nd	17.95	17.95
*Pipistrellus kuhlii*	nd	nd	0.00	2/27	nd	7.41	6.90
*Hypsugo savii*	nd	nd	nd	6/19	nd	31.58	31.58
*Eptesicus serotinus*	nd	nd	nd	20/73	nd	27.40	27.40
*Eptesicus isabellinus*	nd	0/3	0.00	nd	nd	nd	0.00
*Plecotus austriacus*	nd	nd	nd	19/74	nd	25.67	25.67
*Nyctalus leisleri*	nd	nd	nd	0/9	nd	0.00	0.00
*F. Miniopteridae*							
*Miniopterus schreibersii*	7/63	nd	11.11	130/842	0/45	14.66	14.42
*Miniopterus* complex	nd	7/103	6.80	nd	nd	nd	6.80
*F. Molossidae*							
*Tadarida teniotis*	nd	nd	nd	23/105	24/254	13.09	13.09
Total	12/238	30/455	6.06	421/2024	51/585	18.09	15.56

^a^Number of seropositive individuals/number of analysed individuals

^b^Percentage of seropositive individuals per species

Blood samples (30–250 μL, depending on the bat’s size, and always less than 1% of the body mass) were obtained by a small puncture made next to the proximal epiphysis of the radius. The volume of blood was extracted in compliance with FAO guidelines. Vials containing blood were stored at 4 °C for a few hours. Samples were centrifuged for 20 min at 12,000 rpm, and sera were extracted with a micropipette. Serum samples and clot pellets were frozen at −20 °C until analysis. The technique used to detect lyssavirus neutralizing antibodies was an adaptation of the rapid fluorescent focus inhibition test (RFFIT). Titers were expressed as the arithmetic means of two independent repetitions. Samples were considered positive when the number of fluorescent foci was reduced by 50% at the 1:81 dilution, as previously described^11^. Bats that were found dead were collected in the field work. Brain samples were obtained and stored at –80 °C. RNA was extracted from brains and tested by nested real-time polymerase chain reaction (nRT-PCR)^11^.

To our knowledge, this is the first work to report the result of an active lyssavirus survey carried out in bats from Northern Africa. Of the 693 sera analysed, 42 (6%) tested positive for EBLV-1–neutralizing antibodies, and these results were compared to those obtained from two distinct surveys carried out in Spain and Italy^12,13^. Four of the nine selected locations (44%) in North Africa harbored seropositive bats: one in Morocco and three in Algeria (Table 1). EBLV-1 neutralizing antibodies were detected in five (45%) bat species belonging to three families, and 42 of the 693 (6%) samples were seropositive (Table 1, Supporting Information S1).

Three of the seven bat species analyzed in Morocco were seropositive. *Rhinolophus ferrumequinum* was the Moroccan species with the highest percentage of seropositive individuals (12%). Four of the seven bat species sampled in Algeria tested seropositive, and *M. schreibersii* was the species which had the highest percentage of seropositive individuals (11%) (Table 1). When we compared these percentages with those generated from the studies carried out in southern Europe, we observed that 14 out of 17 (82%) Spanish bats and three out of seven bat species (43%) tested in Italy were positive for EBLV-1. The bat species with the highest percentage of seropositive samples were *M. myotis* and *M. blythii*, respectively (we considered bat species with a sample size higher 30 individuals). In Europe, 472 (18%) of the 2609 sera tested were positive (seroprevalence by species ranged from 7.41 to 50%).

In addition, we analyzed 19 brains of bats (11 of *R. ferrumequinum*, 1 of *Rhinolophus euryale*, 6 of *Miniopterus* complex and 1 of *M. punicus*) from three different sites on the Atlas region of Morocco. All samples tested negative for lyssavirus RNA.

The specificity of our RFFIT method had already been demonstrated in a previous study^11^ in which a selection of sera from five bat species using the EBLV-1, EBLV-2, and CVS strains had been tested. All sera were positive against EBLV-1 but negative for CVS and EBLV-2; furthermore, the presence of EBLV-1 seropositive bats in Spain, Italy, Morocco and Algeria provides evidence that EBLV-1 may be circulating on both shores of the Mediterranean Sea. Unfortunately, all samples tested by nRT-PCR were negative, which is not surprising considering the relatively reduced number of samples collected and the low expected prevalence of such viruses in bats. While there is no evidence of bat migration between Africa and Europe, virus transmission could be ascribed to the capacity of bats to fly very long distances. Indeed, *M. schreibersii* seems to play an important role in the maintenance of EBLV-1 in some bat colonies of the Balearic Islands^14^ and in Catalonia^15^. In the Mediterranean regions, *M. schreibersii* is highly abundant and very likely contributes to virus dispersion^12^, as it is able to fly and easily cover the distance across the Gibraltar strait (14 km). Similarly, Sicily is the Italian island where seropositive bats were sampled, and its distance from the Tunisian coastline is approximately 142 km. Menorca and Mallorca (islands belonging to the Balearic Islands, Spain) are approximately 170 km from the east coast of the Iberian Peninsula and 270 km from the coast of Algeria. Therefore, further studies are needed to better understand the link between lyssaviruses circulating in Europe and the serological results observed in North Africa.

## Supplementary information

Supplementary Figure 1
