# Supplementary material for: Active sero-survey for European bat lyssavirus type-1 circulation in North African insectivorous bats
Source: Emerg Microbes Infect. 2018 Dec 13;7:213. doi: 10.1038/s41426-018-0214-y (PMC6292898; doi:10.1038/s41426-018-0214-y)

**Supporting Information S1**

**Figure 1.** Geographical distribution of bat colonies analyzed. The black and blue circles correspond to localities prospected with EBLV-1 seronegative and EBLV-1 seropositive bats, respectively.


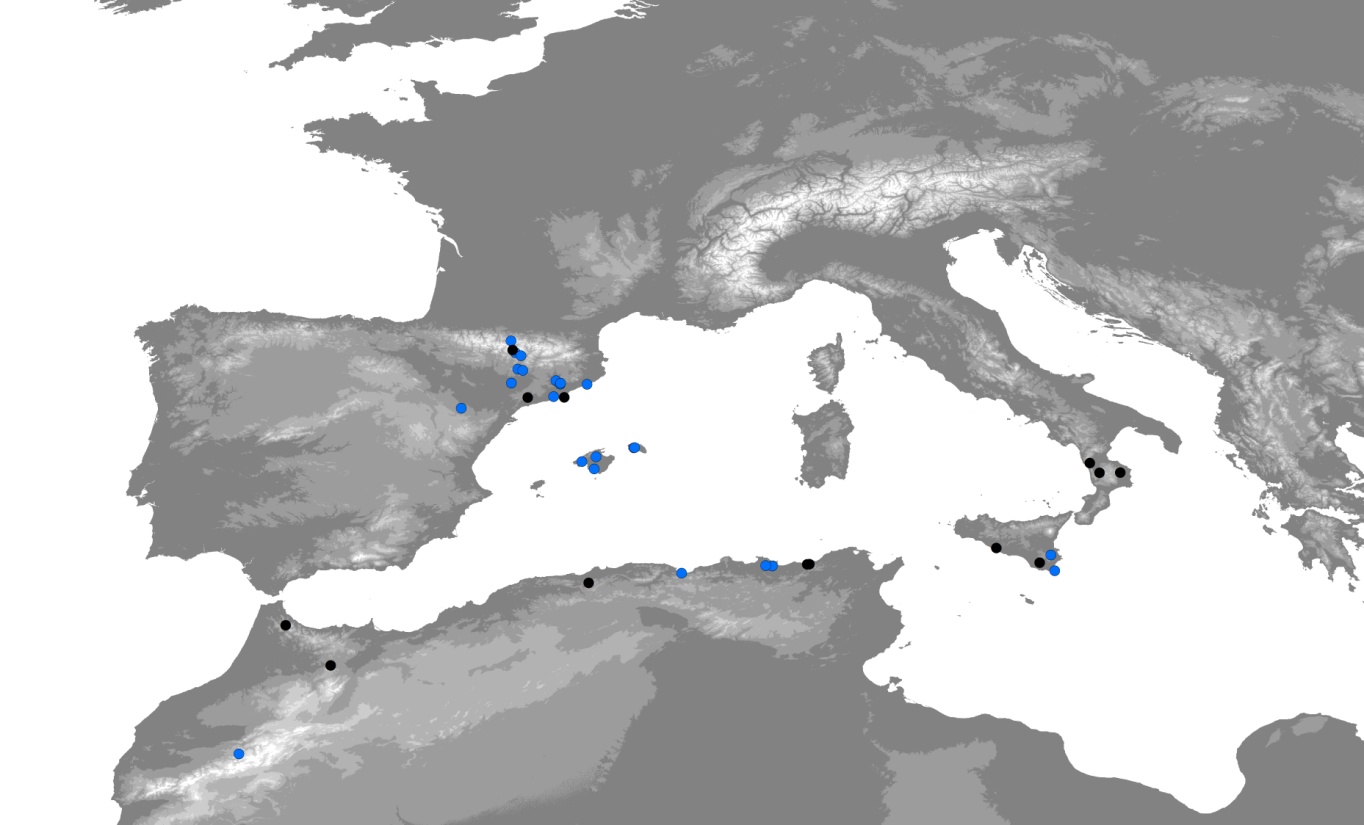

Supplement: Supplementary file 1 — Supplementary Figure 1 [file 41426_2018_214_MOESM1_ESM.docx]
